# Central Persons in Sustainable (Food) Consumption

**DOI:** 10.3390/ijerph19053139

**Published:** 2022-03-07

**Authors:** Carolin V. Zorell

**Affiliations:** School of Humanities, Education and Social Sciences, Örebro University, 701 82 Örebro, Sweden; carolin.zorell@oru.se

**Keywords:** social influence, food choice, climate-friendly eating, social media, political consumerism, children’s health

## Abstract

What people eat has become a highly political issue, closely intertwined with public health, environmental concerns, and climate change. Individuals’ consumption decisions tend to be greatly influenced by the people that surround them, and this seems to be especially true when it comes to food. In recent years, alongside close contacts, such as family and friends, a myriad of social influencers have appeared on the screens, sharing opinions on what (not) to eat. Presenting results from a youth survey conducted in Sweden in 2019 (*N* = 443), this paper shows that social media have become the primary source of *information* about food and eating for youths, followed by schools and families. However, primary sources of *influence* continue to be parents and the family at large. Furthermore, the study shows that it is possible to identify ‘*central persons*’, i.e., relatively clear-cut groups of people whose food choices—measured as tendency to eat climate friendly—is mirrored by the youths, both in their everyday food preferences and in their broader political awareness as expressed through political consumerism. A conclusion from this is that certain people can be particularly successful at inspiring larger numbers of other people to engage with healthier and environmentally friendlier (food) consumption in a society.

## 1. Introduction

What food is consumed, in what way, and with whom, has fundamentally changed over the last decades. On average, today individuals eat more [1], more out of home [2], and more sugary, processed, and in general unhealthy food [1]. This is a major and continuously increasing problem, causing a rise in preventable diseases and premature deaths in countries worldwide [3,4]. Likewise, it is causing great harm to the environment and climate [3,5,6]. The food sector has thus been identified as one of the most important sectors to be urgently changed in order to reach sustainability goals and climate targets [3,5,6].

A key ambition stated across countries is therefore to encourage citizens to choose more ecological, climate-friendly, and healthy food. Especially children and adolescents seem to have been identified as key target group for such endeavour [7,8,9]. They are the adults of tomorrow, and thus whether they develop environmentally- and climate-friendly food habits today will determine the food sector’s long-term success at performing within the Earths’ “planetary boundaries” [10,11].

A common finding is that food choices are related to individual-level factors (e.g., attitudes, tastes, biological and demographic factors) and purchasing contexts (e.g., food availability). It is also increasingly recognised that food choices and behaviours are very much related to what peers and parents eat and drink [12,13], and, in a broader sense, to those sharing a social identity [14,15]. However, in a massively growing media landscape, the sources of information and the ways of communicating about food have changed fundamentally. B/vlogs, YouTube channels, (pseudo-)documentaries, social media posts, and food influencers have mushroomed, spreading recommendations about what to eat or not to eat. Digital spheres seem to have become an ever more important source of information about food, diets, and their implications for both health and the environment, especially—though by no means exclusively—for adolescents and young adults [16,17].

Hence, the number of potential sources of influence on when, where, and what a person eats seems to have exploded. However, just because people get information from certain sources, this must not necessarily mean that it translates into behaviour [18]. Initial studies suggest that digital media influence food choice and intake of children and youths [16,19]. How their influence compares to non-digital sources, such as parents or friends, remains unclear though. A strand of literature in social psychology and economics highlights that certain people have more influence on their peers than others in making them adopt certain attitudes or behaviours. Crucially, these studies suggest that on the basis of certain relationship characteristics or attributes, one can identify such “central persons” [20] or “social referents” [21]. Additionally, having such central persons changing behaviour and disseminating information about it seems to effectively achieve behaviour change of individuals and in larger populations [20,21].

This paper aims to study if such ‘central persons’ can also be identified in the context of sustainable consumption and eating (meaning, ethical, environmentally- and climate-friendly). This is done through addressing three sets of questions which stem from the above-mentioned:

*RQ1: To what degree do digital sources of information about food influence the food choices of youths, and how does their influence compare to that of close ones such as parents or friends?*

*RQ2: Can the idea of ‘central persons’ be applied to the sphere of food, that is, can we identify certain kinds of people that are particularly central to (youths’) everyday food choices and influence what they eat, and if so, how can they be identified?*

From this, a third question follows. As described above, food is tied to great socio-political challenges. Analogous, eating ethical, ecological, and ‘climate-friendly’ food has become political, expressed among other things through so-called political consumerism [22,23]. Additionally, the individual responsibility to be considerate about the wider societal and environmental implications of consumption choices is hotly debated, highlighted, and gradually acknowledged across spheres and people [24]. This raises the question:

*RQ3: Can the behaviours and recommendations of central persons be ‘catalysts’ of a broader political engagement, such as boycotting and buycotting products for ethical, environmental, or other political reasons?*

In keeping with this last question, the paper focuses on environmental and climate aspects of food. To investigate into the various questions, it uses data from a high school survey conducted in Sweden in 2019 (*N* = 443). Next to questions about everyday food habits and sources of information about food, health, and ecology/climate, it contains an original measure to identify ‘central’ persons and their perceived eating habits. However, before presenting the data, methods, and results in detail, the paper starts with a review of current knowledge on the roots of eating patterns and food influence(r)s. Afterwards, it proceeds with presenting the empirical results. The paper concludes by discussing its contribution to the understanding of the role of social influence(r)s in defining eating patterns in the context of acute needs for more ecological and, generally, sustainable food choices.

## 2. Theoretical Background

### 2.1. Food Information and Influence

Individual preferences, attitudes, tastes, biological traits, and demographic backgrounds play a major role in determining when, what, and how much a person eats. Considerations such as convenience, price, taste, certain dietary requirements, health objectives or environmental values can guide what an individual is interested in knowing about food, where they search for information about food, and what they eventually buy and eat [13,25]. However, there are multiple other sources from the social and physical environments which inform and direct individual food choices [26,27,28,29,30,31,32] (see for a review [12]).

Parents play a particularly crucial role. By way of how and what parents provide for eating, what they encourage (not) to eat, and what they eat themselves, parents influence what their children eat, from early childhood throughout adolescence into adulthood [2,33,34,35,36]. Peer groups are another place where individuals discuss, get informed about, and mimic food preferences and habits. What and how much peers eat and weigh, especially close friends, has been shown to be tightly correlated with individual eating habits and weight [32,37,38] (but see also [33]). This points to much time being spent together consuming food, possibly talking about food, and influencing each other’s food habits.

A third important factor shaping where and how food is purchased and consumed is physical and socio-cultural contexts. Such contexts cultivate socio-cultural norms, values, and traditions, and they typically build on certain policy and regulatory frameworks. These shape what is produced and imported in a country [12] and how individuals and media communicate about food (e.g., through advertising laws [39,40]). Thus, they determine what kind of food is made available, in which quantities, and what is socially accepted and encouraged to buy and eat (e.g., [40]). Furthermore, cultural belongings and social identities can influence, e.g., customs and tastes, and thus choices [14].

In modern everyday lives, these individual predispositions and immediate social and physical influences compete with multiple other aspects. In print and digital media, schools, and other outlets, individuals encounter a wide array of news and information about impacts of food on health and the environment. Some advocate certain diets for reasons of, e.g., attaining a certain body shape or look, or on how to ‘comfort’ the self through food. Others connect the advertised foods or diets to certain social images and groups [41], conveying the impression that for becoming or remaining a certain person or part of a social group, one should eat this or that [42]. Simultaneously, food advertisements surround individuals everywhere, most commonly for snacks and food that can be considered unhealthy [19,43,44,45]. Given their usually processed and plastic-packaged nature, they typically cannot be considered environmentally friendly either. Taking the multitude of messages about diets and lifestyles together, they are often contradictory and give rise to conflicting recommendations, desires, and needs.

As it becomes difficult to ascertain what food choice is the ‘right’ one, individuals find themselves in recurrent situations of uncertainty. Here, they likely look for orientation. Numerous studies show that exposure to advertisements, recommendations, and to less obvious marketing of products, especially in social media, has a discernible impact on attitudes towards the products and the likelihood to consume them [42,46,47], see also [12] (p. S66). This is found even when individuals disapprove or are unwilling to acknowledge such influence [47]. The interaction between different users in their roles as individuals seems to be an important driver of such influences [48,49]. In a recent randomised experimental study, Coates and colleagues [16] show how seeing social media influencers that eat unhealthy food successfully purports consumption of unhealthy food (measured in terms of calories) among children. Other research comes to the same conclusion. Media and advertisements, including user-generated content, predominantly focus on food that can be considered unhealthy; and, as a whole, it is mainly the intake of unhealthy food which is purported *successfully* in (social) media [43,50]. The information and messages surrounding an individual in these spheres thus seem to serve as anchors that give orientation in the decision what to eat.

Research yet also suggests that for being granted attention and successfully influencing an audience, individuals, brands, and their relationship towards a person need to satisfy certain criteria. For instance, they need to be able to raise feelings of accessibility and a “warm personal relationship” [49] (p. 5). Additionally, the ability to establish and maintain a trusting relationship seems to be of crucial importance [51], which can enable and be enabled through interpersonal contacts [52]. Correspondingly, personal closeness (family, close friends) and trust in the counterparts’ competence in food matters (schools, teachers, doctors) play a key role in developing food preferences and choice [35,53].

However, social media very much build on such principles, too. Certain styles of language and images are used to raise feelings of closeness and shared social identities, with the ambition to influence peers and followers. Groups are created (e.g., in Facebook) or imagined (e.g., the following community of an Instagram influencer) and some seem to influence thousands (or millions) of followers to adopt certain diets, try recipes, or purchase certain (food) products.

A general contention thus is that social media is increasingly outdoing parents and other close ones as sources of information and influence about what to eat, especially for young people. Yet, how the various ‘channels’ of information and potential influence fare compared to each other remains largely uncharted. The knowledge gap is even greater when one includes other sources that spread information about food, health, or climate-friendly eating, such as TV, magazines, and civil society actors. One may agree with the contention and expect that (hypothesis h1) *social media have become the most important source of information about food*. Yet, parents, schools, and friends are the daily interaction partners of youths. An alternative hypothesis thus is that (ha1) *parents, friends, and schools are the most important sources of information about food*.

### 2.2. Central Persons and Food Habits

Transmitting information to people does not necessarily entail getting them to do something [54]. Some researchers studying social influence and imitation differentiate between different kinds of persons and strengths of influence. As they highlight, some persons are more influential—or ‘central’—in opinion and behaviour formation than others are [21,54,55,56,57].

Crucially, Banjeree and colleagues [20] show that it is possible to identify concrete persons in a community who are better at spreading messages (e.g., gossips) and calls for action (i.e., getting people to get inoculated). Similarly, Paluck and her colleagues [21,56,58] could identify certain individuals that were more apt than others at instigating changes in attitudes and behaviours (in their case, harassment behaviour at schools) by changing perceptions of collective norms. Food consumption and the development and change of food preferences and habits tend to occur in social settings. Hence, the food individuals choose in their everyday lives may be similarly influenced.

Importantly, observing ‘central persons’ engaging with aspects such as ecology and climate change in relation to their food choices could motivate an individual—be it an adolescent or an adult—to do the same. Central persons can work as a source of information about food, food habits, and food norms in general, as well as about sustainable and climate-friendly eating in particular. In conversations and as living examples, they may inspire others to reflect upon their food habits, and to try and adopt new ones. In other words, observing central persons engaging with sustainable consumption and eating, e.g., eating climate friendly, could be an effective way through which more people get drawn into sustainable consumption.

Hence, the insights by Banjeree, Paluck, and their colleagues suggest that some people may be more powerful at influencing others’ food preferences than others, and with this, at drawing people into sustainable eating. Hence, hypothesis 2 is that (h2) *it is possible to identify certain kinds of persons that are more ‘central’ than others at influencing people’s food preferences.*

Moreover, this could work as an entry point into broader engagements with politics. By raising interest in the political implications of food, it may stir a broader socio-political awareness. In this vein, political scientists have consistently shown that political participation—mainly studied in terms of voting—is connected to what surrounding people say and do (e.g., [59,60,61,62,63]). Correspondingly, what central persons eat may not only affect individual food preferences. If individuals perceive their central person focus on political issues such as climate friendliness of food, they may become interested in politics in a broader sense. That is, they may engage with the politics of consumption, e.g., through deliberately choosing to buy or boycotting certain foods, diets, and other products for ecological, ethical, or other political reasons [64,65]. Put simply, a last hypothesis is that (h3) *an individual’s boycotting and buycotting engagement correlates with the perceived tendency of their central persons to ground food choices on political considerations.*

## 3. Materials and Methods

The subsequent empirical study uses data collected as part of a survey of Swedish pupils in their last semester at senior high in 2019. The selection of schools was guided by a combined sample strategy of strategic sampling, where the aim was to cover classes with different subject specialisations (natural sciences, social sciences), orientations (aiming for further education or practical work after school), and from towns of different sizes in mid-Sweden (five in total, ranging from Stockholm as a big city, to Hallsberg municipality as a small town area). Yet, regardless of this strategic approach, the sample needs to be seen as a convenience sample.

Before the survey, the teachers made available the information about the study aim and ethical issues in their school’s internal information system, so the pupils had time to go through the material. Data collection took place in the classrooms, for anonymity and quality reasons without the teachers. Trained study assistants distributed again information about the study and ethics and collected informed consent before students would receive a link to the electronic questionnaire (a few answered in paper format). The assistants remained in the classroom while the students filled in the questionnaire, which took about 90 min (with a break in between). The students did not get any reward for answering the questionnaire. Those who did not want to participate did schoolwork instead. The final sample consisted of 443 pupils aged 16–22; this excludes ca. 40 respondents who provided answers that gave reason to suspect systematic misreporting. In total, 59% of the respondents were girls, 39% boys, and 1% youth identifying with another gender.

To measure the most important *source of information* (hypothesis 1), the youths were asked from where they usually get information about food, eating, and ecology/climate. To this end, they were presented a list of six kinds of social ties and media (family, friends, school, social media, TV, newspaper) plus an open answer field and asked to assign to each of them a rank describing to what extent they usually get information about food from that source. Given the setup of the questionnaire, the participants assigned to each of the seven potential sources of information a number between 1 (least usual) and 7 (most usual). This resulted in seven variables with seven categories, i.e., scale values. For each value, the frequency of responses indicates the number of people who ranked the source of information on the respective rank. Comparing these frequencies permits drawing conclusions about the comparative importance of the distinct kinds of social ties and media as sources of information for the youth. However, in many cases, the ranking was not entirely ‘neatly’ done, but respondents assigned same ranks to more than one source and no source to single ranks. Since the focus of measurement is on identifying the *most important* source of information, the analysis focuses on the frequency to which each source was ranked first (i.e., as main/most important); if single individuals ranked more than one with “1”, all of them are counted as “most important”. This renders a comparative assessment of the various sources’ importance.

The dependent variable for hypothesis 2 grounds on a validated questionnaire capturing *everyday food preferences.* It asks respondents what is important to them that the food they eat on a typical day fulfils. Through 24 response items, individuals can indicate on a 7-point scale whether a trait is not at all (1) to very (7) important to them [66,67,68]. The items include price, various health-related, sensory, and popularity aspects, as well as ecological and ethical criteria. A factor analysis provides five distinct factors, of which one discernibly relates to ‘sustainable’ eating (Cronbach’s α = 0.934; see Appendix A). It covers that the food should be organic, fair traded, produced and packaged environmentally friendly, be in harmony with one’s ethical values, and climate friendly. On its basis, an additive index is created that captures the degree to which an individual puts weight on the fact that their everyday food choices are ‘sustainable’.

To capture *central persons*, a measure was adapted from Åmna and colleagues [69] (see Appendix B). Respondents were asked whether certain kinds of persons have a greater influence on their food choices than others, and if so, to freely mention one or two such persons. To make the question simpler to understand, the wording further specified “who this person is, for example a person [they] follow on social media, [their] best friend, [their] mom or dad, a celebrity, the leader of an organization”.

As a second independent variable (*climate-friendly eating of central persons*), the youths were asked if and to what extent one or the two of these persons themselves “buy/eat climate-friendly food”. Here, the answer options included (1) “No, they do not”, “Yes, one does so (2) sporadically/(3) most of the time”, “Yes, the two do so (4) sporadically/(5) most of the time”, as well as a (6) “I do not know” (see also Appendix B). Despite the question referring to the prior question, examining the data revealed that the youths did not necessarily refrain from answering this question despite previously having answered that there is no central person. In total, 19 individuals answered that one or two central persons are eating climate friendly, despite not having named any central person beforehand. This might be due to the additional effort that writing down a central person implies (first question), whereas ticking a box (second question) requires less cognitive effort. A check showed that these responses do not change the general conclusions. Nonetheless, in the following analysis the two variables are treated as separated from each other.

For hypothesis 3, two questions gauged the regularity of political consumerism. Adapted from commonly employed measures [63] (p. 9), they ask how often it occurred in the last 12 months that the person (1) boycotted and (2) bought food or other products for ecological or ethical reasons. Respondents then indicated on a 6-point scale whether they did so never (1) to every day (6). The *boycotting* and *buycotting* variables are correlated (*r*_p_ = 0.546; *p* = 0.000; *N* = 432), yet there are differences across individuals and thus the two are considered as separate dependent variables.

Finally, *age* (measured in years of age) and *gender* (girl (0), boy (1), other (0)) were included in all models as control variables. Table 1 summarises the descriptive statistics for all variables.

The methodological approach starts with descriptive and correlation analysis. This is combined with non-parametric group tests (Kruskal–Wallis H test) to compare groups of people with and without central persons that eat in a certain way (i.e., climate friendly).

## 4. Results

### 4.1. Identifying Central Persons Influencing Food Preferences

**Figure 1:**
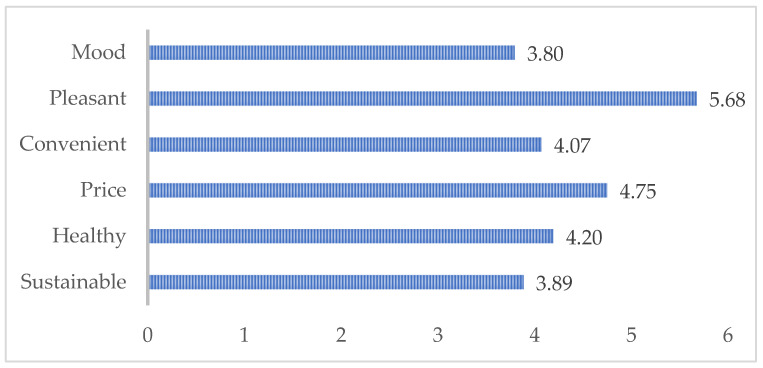
Food properties guiding food choice of youths, mean values.

To map the terrain, the enquiry starts with a look at the principle factors that the youths mention as guiding their food choices. Figure 1 reports the mean values for the five overarching categories (which, according to a non-parametric Friedman test, are statistically different from each other; *χ*^2^(4) = 492.77, *p* < 0.001). The principal factor are sensory and emotional aspects, i.e., taste, texture, and mood. This is closely followed by price and, with some distance, health criteria. All three are not different for girls and boys. For the third gender, given the small number of respondents, a discussion would be speculative in character and is therefore excluded from this comparison. 

Ecological criteria range lowest in influence on food choice. However, they are notably different between girls and boys. Girls show a greater tendency to consider sustainability important than boys. This difference fits in with multiple other studies on environmental behaviours, showing that across all age groups, women tend to be more considerate of ecological and ethical consumption aspects than men (even though the gap has narrowed over the past years; cf. [70] p. 132). Overall, however, it seems clear that the issue of food choices remains complex, and the youths are far from all being “Greta”, that is, young environmental activists who have changed their lifestyles entirely.

**Figure 2:**
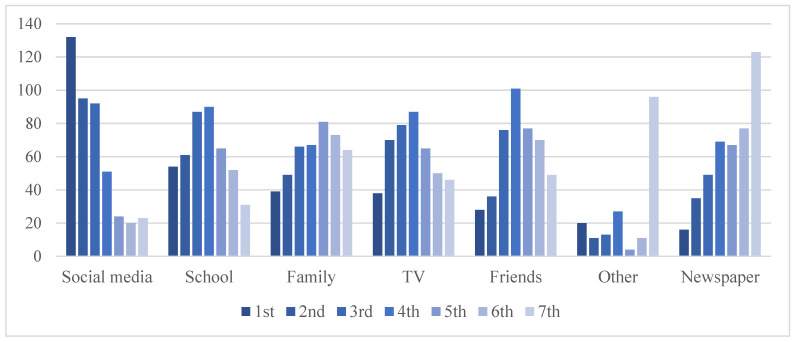
Most usual sources of information about food (numbers of respondents ranking source 1st to 7th). Note: Ordered from left to right according to source that was most to least often ranked as most important.

Such differences in the priorities can be rooted in various kinds of personal circumstances. A main one as suggested in this paper is where individuals get information about food and the people with whom they interact discussing food-related matters. Looking into this question, Figure 2 summarises the ranking provided by the respondents of the sources through which they usually receive information about “food, eating, and climate matters”. Their comparative ranking is interpreted as an indication of their comparative importance; that is, the sources were the youths state to get information most (usually) are here understood as being the most important sources of information.

Visibly, social media headed the ranking with a mean value of 5.16 on a 7-point scale. With notable distance, this is followed by the family and school. After this, TV and friends follow. Newspapers and other sources rank as least usual sources of information. Some respondents also specified what they mean with “other” sources; those named by several respondents include statistics, (online) documentaries, and podcasts. A single respondent named politicians. According to a non-parametric Friedman test, these mean values are different from each other to a statistically significant extent (*χ*^2^(6) = 189.87, *p* < 0.001).

Digital media outlets, especially social media, are thus the principal ‘channel’ through which youths receive information about matters relating to food. This confirms hypothesis 1. Of the non-remote sources, schools, teachers, and the family can be considered important, while friends less so. Overall, however, they do not seem to be the most important sources of information about food, which disproves the alternative hypothesis (ha1).

Influencers on social media and teachers at school may advocate certain healthy diets. However, this does not automatically and necessarily mean that the receivers of the messages adopt their dietary recommendations. This leads to the question whether there are certain kinds of people who are particularly central at *influencing* a person’s everyday food choices. This can be gauged in two ways. By directly asking about the existence of central persons; and indirectly, by observing if a potentially central person has an influence on what a person eats.

The survey includes a direct question. Furthermore, while it provides only cross-sectional data, which does not allow for studying a causal connection between eating patterns, the questionnaire includes questions that allow for comparing the eating patterns of a group of youth whose central persons eat climate friendly with that of a group whose central persons do not eat climate friendly. This way, we can study if the patterns of climate-friendly eating of central persons correspond with the eating patterns of the respondents.

About 38% of the youth state that there is a specific person that has especially affected what they buy and choose when it comes to food. Correspondingly, a majority states that they *cannot* name anyone that has a central role in influencing their food choices. While this does not necessarily mean that there is no one affecting their choices, it yet means that they have no one who consciously and distinctively affects them, and who can be named off the top of their head.

**Figure 3:**
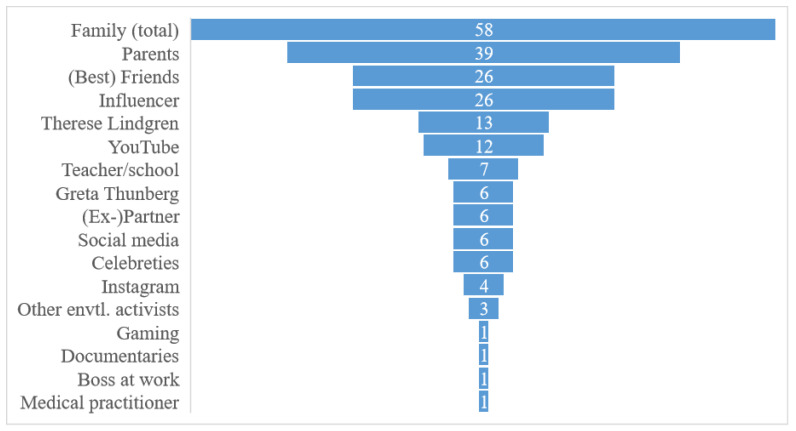
Central persons affecting food purchase and choice, frequencies of mentions. Note: Family (total) includes parents plus any other kind of family tie. Therese Lindgren is a Swedish influencer on lifestyle issues in general. The figure reflects the responses provided by 38% of participants, i.e., 166 individuals. In total, 12 persons provided answers that could not be classified. A total of 89 individuals named one central person, the rest named two and a few named a combination of several categories in relation to a single central person, e.g., Therese Lindgren *and* YouTube *and* influencer; in the graph, these cases are counted, respectively, with a value for each of the different categories.

Figure 3 reports the sorted answers to the open question about who the central persons are (i.e., responses given by 38% of respondents). Instagram, influencers, etc., are not directly grouped under the label of social media to illustrate the variation in answers. Besides, it gives an impression of the various sub-types and variety that the label ‘social media’ actually covers. 

The graph renders a relatively clear picture, which slightly diverges from the results regarding sources of information. When it comes to actual influence on food choice, it seems that family members, and especially parents, range similarly high in importance as social media, even after subsuming the different sub-types of social media into one category. Hence, parents may not be considered primary sources of information about food, but they are primary sources of influence. Admittedly, this is not entirely surprising as most youths live at home (91% of the respondents), and hence may find their parents choosing what food is bought and served to begin with. This also likely influences the food tastes and habits in situations where the youths make choices on their own. However, parents are also central for those who do not live at home, thus pointing to an additional kind of influence besides that of owning the decision-making authority.

Best friends are important too, ranging on the same level as influencers. Schools and teachers, in turn, seem to be important sources of information, but according to this descriptive result, they appear to lack influence on what is eaten.

Table 2 reports the responses to whether one or two central persons eat climate friendly. A clear majority reports perceiving at least one doing so. What is more, they are perceived to be doing it more regularly than sometimes. This is surprising, since it suggests a disproportionately large fraction of people eating climate friendly. An alternative reason may be that the respondents observe it consciously precisely *because* it stands out. In any of the two cases, the figures should not compromise the later conclusions, but they will be kept in mind when drawing general conclusions from the results.

According to the theory of central persons, if an individual perceives that their central person(s) focuses on something such as the climate friendliness of what they eat, the individual would be expected to reproduce it in their everyday food choices. To test this theory, one can look at the overlap between the frequency with which an individual perceives their central person(s) is (are) grounding food choices on certain aspects (i.e., climate-friendly eating) and the individual’s own frequency of doing so. The variable assessing perceived central persons’ climate-friendly eating is treated as an ordinal variable in the analysis, i.e., the influence of two persons eating sometimes climate friendly is considered to be stronger than one person doing so regularly. This assumption is based on research into social influence and behaviour spread, suggesting that several influences tend to reinforce each other and therewith strengthen the influencing effect [54]).

The test statistics of a Kruskal–Wallis H test indicate that there is a statistically significant difference in the median focus that respondents put on sustainability aspects of their food depending on the degree to which they perceive their central persons to be doing so (i.e., the independent variable as reported in Table 2) (*χ*^2^(4) = 24.934, *p* < 0.001). Hence, the youths’ focus on sustainable eating seems to indeed vary along with what they perceive their central persons are doing. Non-parametric correlation analysis further specifies that what central persons are perceived to be doing is *positively* related to what the youths themselves do (*r*τ = 0.235, *p* < 0.001, *N* = 194). However, when differentiating according to gender, the effect is again different among girls and boys. For girls, the correlation coefficient does not reach statistical significance (*r*τ = 0.099, *p* = 0.163, *N* = 120), while for boys it is moderately strong (*r*τ = 0.312, *p* < 0.001, *N* = 69).

**Figure 4:**
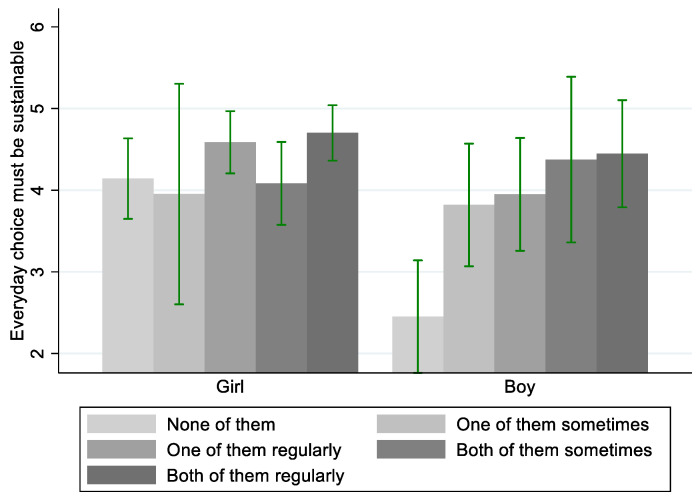
Central persons and youths eating climate friendly. Lines show 95% confidence intervals.

A bar chart visualises this difference (Figure 4). Girls’ inclination to consider it important that their everyday food choices are sustainable is high throughout. The mean values are slightly higher among those reporting that one or two of the central persons are eating climate friendly on a regular basis. Yet, the confidence intervals overlap with the other categories. Thus, their inclination seems to be relatively independent of what their central persons are perceived to be doing. In contrast, boys who do not perceive any central person to be eating climate friendly do not either. Yet, if one or two central persons sometime or regularly eat climate friendly, they tend to do so as well. Moreover, the inclination increases quasi-linear along with the increased number and frequency of central persons eating climate friendly (although the confidence intervals overlap, suggesting that this last interpretation must be taken with caution).

In sum, the results suggest that there are central persons. These central persons seem to belong to relatively clear-cut groups of people, who can be characterised by a distinct type of affiliation to the individual (e.g., family, friends, social media influencer). Yet, perceived centrality does not necessarily mean behavioural centrality. For girls, influence seems to happen mainly if central persons eat climate friendly on a notable, i.e., regular basis. Boys, in turn, the more frequently they perceive their central persons to be grounding food choices on climate considerations, the more frequent they ground their everyday food choices on such criteria as well. This association can be interpreted as a potential influence coming from the central person, which allows for confirming hypothesis 2.

### 4.2. Central Persons and Political Consumerism

The last hypothesis (h3) states that what central persons are perceived to be doing can affect more than eating habits. If they are perceived to be grounding food choices on climate considerations, this may get the ones to whom they are central to engage with the politics of food and consumption more widely. In other words, central persons’ behaviour may instigate broader political awareness and motivate political engagements, especially political consumerism. Two dependent variables are used to measure such engagement: boycotting and buycotting. The independent variable is the degrees of climate-friendly eating of central persons.

The results of a Kruskal–Wallis H test indicate that there is a statistically significant difference in buycotting between the youth within the five different degrees of perceived climate-friendly eating of their central persons (*χ*^2^(4) = 43.370, *p* < 0.000). Non-parametric correlation analysis further points to a positive relationship, for both girls (*rτ* = 0.212, *p* = 0.006, *N* = 120) and boys (*rτ* = 0.431, *p* < 0.000, *N* = 67). For boycotting, the group test also reveals a statistically significant difference between the five degrees of what central persons are perceived to be doing (*χ*^2^(4) = 19.122, *p* < 0.000). Yet, according to correlation analysis, this holds only for boys (*rτ* = 0.273, *p* = 0.006, *N* = 66), while not for girls (*rτ* = 0.100, *p* = 0.187, *N* = 120).

**Figure 5:**
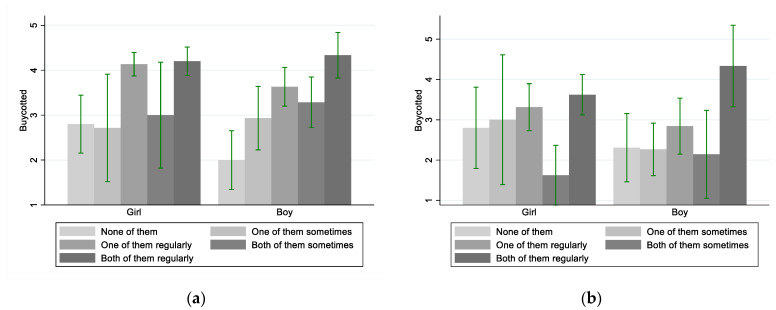
Central persons and youths’ political consumer engagements, (**a**) buycotting and (**b**) boycotting. Lines show 95% confidence intervals.

Figure 5 specifies the patterns. Girls’ tendency to buycott is connected to central persons’ engagement with climate-friendly eating if the central persons do so regularly, but not if they are perceived to be doing so only sometimes or not at all. An explanation may be that the baseline for girls is higher as they have generally a higher tendency towards being already engaged with the politics of food and consumption. Hence, they may note and be influenced only by those cases that stand out through great regularity of climate-friendly consumption. Girls’ boycotting engagement does not appear to be related to what their central persons do.

Conversely, boys’ engagement with buycotting is more closely related to what their central persons are perceived to be doing. If they are perceived to never eat climate friendly, the boys do the same. If they are perceived to focus on climate-friendly eating sporadically or frequently, the boys respectively buycott sporadically or frequently. Boycotting, however, seems to need a stronger engagement on the central persons’ side to uncover an effect. That is, a potential influence seems to be observable only if central persons, be it one or two, are perceived to *regularly* engage with climate-friendly eating. Overall, it seems reasonable to confirm the part of hypothesis 3 relating to buycotting. The youths’ buycotting engagement is closely related to what they observe of their central persons, and this is especially strong for boys. Boycotting, in turn, seems to be related only for boys and only provided central persons send strong signals through regular engagements with the politics of eating.

## 5. Conclusions

The consumption of unhealthy and unecological food is a major and continuously increasing problem, causing a rise in preventable diseases, premature deaths, and ecological disasters in countries worldwide [3,4,5,6]. With the ambition to contribute knowledge to advance ambitions to get citizens choose more ecological food, this paper provides insights which suggest that gaining certain kinds of people adopting climate-friendly diets can act as multiplier inspiring other people to do the same.

In a study of Swedish high school students, the paper could identify relatively clear-cut groups of people who show to be particularly central for what youths prefer to eat. These groups are characterised by a distinct type of affiliation to the individual: the family, friends, or influencer on social media. Depending on whether these central persons are seen to eat climate friendly or not, the youth respectively consider environmental and ethical aspects important in their food choices as well, or they do not. This influence of central persons seems to be particularly great on boys, and although to a lesser extent, also on girls. Moreover, their influence seems to extend beyond everyday food habits and come along with broadened political awareness that is expressed through political consumerism.

From a more general perspective, adults tend to like to see themselves as less vulnerable to others’ influences, yet research suggests otherwise (e.g., [48,71]). Hence, while these findings rest on a youth sample, we can expect that similar tendencies would be observable in other age groups. Nonetheless, a limitation is that the youth may not always buy food for themselves and need to eat what is served. This can imply that we are observing a larger influence of especially parents in this study as what would be observed in a study of the general population. In addition, it carries the possibility that there are various kinds of influence observed in the present study, again especially of parents on their kids, as they may not only ‘inspire’ their kids towards certain food choices, but also choose the food they get to begin with. Future studies, therefore, need to explore more in detail what happens within families, and to what extent the influence of the family is temporary.

The findings illustrate two other things. First, there seems to be an important distinction between sources of information and sources of influence. Schools and teachers, for example, are important sources of information for the youth, while they seem to have little actual influence on food choice. The reverse holds for parents and friends. Social media, in turn, fare high on both aspects, but more so on the provision of information.

A common contention is that social media are more relevant in youths’ lives than in that of adults. However, the findings suggest that even among the youth, social media influence seems to be on average lower than that of family and friends. This allows for concluding that findings regarding influence may be quite similar in a study of the general population. That is, close ties such as family and friends are principal influencers. This includes the possibility of a recursive link where youths influence their parents.

The findings further suggest that to successfully promote changes in diets, it is important to focus on addressing and convincing those who can actually influence others to change what they eat, i.e., *central persons*, rather than only disseminate information through, e.g., leaflets and educational programmes. The paper also provides a methodological tool for it: one can identify certain kinds of ‘central’ people based on easily observable characteristics (e.g., being a parent or a v/blogger). Hence, knowing about such ties can work as a methodological shortcut to identify persons who can influence eating patterns particularly well. Campaigns and broader strategies aimed at promoting ecological and healthy eating may then not need to start each time by categorising and assessing each individual’s very personal conception of their social influencers. Instead, they can build on those easily identifiable relationship characteristics and explicitly target those groups, who can act as multipliers.

Second, the observations underscore how supposedly small everyday actions such as food activism can multiply and trigger broader engagements with the politics of consumption, climate change, and political issues more in general. Thinking one step further, this process may not only unfold at the level of the individual who engages in gradually more ways with such issues, but also at the level of a society, where engagements of some can ‘tip over’ to others, inspiring them to engage with the politics of, e.g., food and climate change. Future studies may apply social network approaches to dive deeper into this idea and study whether the influence of certain people can especially effectively advance sustainable consumption in a larger population.

These future studies may then also cover aspects which represent limitations of this study: the cross-sectional nature of the data, the small sample size, and the focus on youths and only one country. Additionally, there is the possibility of reporting bias due to the potential desire of respondents to put themselves in a better light when reporting what they care for and tend to consume. Future studies may include more systematic measures to control for such biases. Likewise, they may focus on further age groups (also to study who are the central persons of those central persons) and include more socio-demographic and economic background variables, as well as longitudinal measures.

This paper made a first effort to compare roles of different kinds of people in the process of changing consumption patterns. It reconfirms that eating is deeply connected to social environments. Yet, it adds the insight that not all social ties are equally relevant. Additionally, while the findings are based on a youth sample, there are reasons to expect that it also applies to adults. This is an invaluable insight for those aiming to transform current food consumption patterns. Specifically, gaining central persons for the purpose may be a key to reach social tipping points at which ‘sustainable’ consumption choices become the ‘new normal’.

## Figures and Tables

**Table 1:** Descriptive statistics for dependent and independent variables.

Variable	Obs.	Mean	Std. Dev.	Min	Max
Additive index everyday food choices must be sustainable	442	3.89	1.41	1	6.78
Boycotting	432	2.78	1.76	1	6
Buycotting	439	3.39	1.29	1	6
Source of information:					
Family	430	4.15	1.80	1	7
Friends	429	3.52	1.68	1	7
School	429	4.03	1.72	1	7
TV	428	3.66	1.73	1	7
Newspaper	428	2.83	1.71	1	7
Social media	431	5.16	1.77	1	7
Other	162	2.78	2.29	1	7
Central person(s)	432	1.38	0.49	1	2
Climate-friendly eating of central persons	194	3.33	1.40	1	5
Gender	436	0.40	0.49	0	1
Age	440	17.92	0.66	16	22

**Table 2:** Central persons eating climate friendly.

Central Persons Eating Climate Friendly	Frequencies	Percentage
None of them	27	13.92
One of them sometimes	23	11.86
One of them regularly	66	34.02
Both of them sometimes	15	7.73
Both of them regularly	63	32.47
Total	194	100

Note: Next to these 194 individuals and the ones who said they have no central person, another 46 individuals answered with “I do not know”.

## Data Availability

The data presented in this study are available on request from the corresponding author.

## Appendix A

**Table A1:** Factor analysis for important aspects in everyday food consumption. Principal Component Analysis. Varimax with Kaiser Normalization. Only loadings > 0.400 are shown. KMO: 0.906, Chi-sq.: 5,484,406. Explained Variance: 69.40%, Cronbach’s Alpha: 0.934, 0.895, 0.731, 0.492, and 0.431, for components 1–5, respectively. Components 1 and 2 are moderately-strong correlated (0.504), while all other correlations range between 0.043 (components 1 and 4) and 0.293 (components 2 and 5).

Component						
	Sustainable	Healthy	Price	Convenience	Sensations	Mood
Produced in an environmentally friendly way	0.894					
Climate friendly	0.885					
Packaged in an environmentally friendly way	0.868					
Environmentally friendly	0.849					
Organic	0.848					
Fairtrade	0.810					
Animal friendly	0.712					
In line with my ethical values	0.661					
Natural	0.469					
Nutritious		0.827				
Much protein		0.816				
Keeps me healthy		0.809				
Much fibre		0.806				
Vitamins and minerals		0.780				
Good for the teeth/ hair/nails etc.		0.690				
Healthy		0.639				
Helps me control my weight		0.506				
Not too expensive			0.851			
Affordable			0.833			
Well-known				0.805		
Convenient/simple				0.649		
Pleasant sensations					0.805	
Tasty					0.739	
Mood						0.869

## Appendix B

Questions gauging central persons:

Q1: Is there someone who has particularly influenced what you buy and choose when it comes to food and drink? If so, please state who this person is, e.g., a person you follow on social media, your best friend, your mom or dad, a celebrity, the leader of an organisation.

Please specify one or two of the most important persons.

0 = No one

1 = Yes, my (1) __________________________

(2) __________________________

Q2: If you have specified at least one person, does any of them buy/eat climate friendly food?

1 = No, they do not

2 = Yes, one does so sporadically

3 = Yes, one does so most of the time

4 = Yes, the two do so sporadically

5 = Yes, the two do so most of the time

6 = I do not know
